# Weight Bias Education in Medical Training: Insights from Stakeholder Perspectives

**DOI:** 10.1007/s40670-026-02731-6

**Published:** 2026-04-24

**Authors:** Tracy L. Oliver, Lisa K. Diewald, Stephanie Felgoise, Austin Kanyuh, Renata Brito-Cherrin, Margaret Brace, Gail E. Furman, Rebecca Shenkman

**Affiliations:** 1 M. Louise Fitzpatrick College of Nursing, Villanova University, 800 E. Lancaster Avenue, Villanova, PA 19085 USA; 2 M. Louise Fitzpatrick College of Nursing, MacDonald Center for Nutrition Education and Research, Villanova University, Villanova, PA USA; 3 School of Professional & Applied Psychology, Philadelphia College of Osteopathic Medicine, Philadelphia, PA USA; 4 College of Liberal Arts and Sciences, Villanova University, Villanova, PA USA

**Keywords:** Weight bias, Medical education, Obesity, E-learning, Qualitative analysis

## Abstract

**Introduction:**

Weight bias among physicians remains a significant barrier to equitable healthcare for patients with obesity. One possible contributing factor is the absence of a structured curriculum addressing weight bias within medical education.

**Methods:**

This project explored medical students’ and faculty members’ understanding of weight bias and identified gaps within the medical school curriculum. Participants’ responses were subsequently used to inform the development of e-learning modules for implementation within a medical school curriculum. Semi-structured focus groups were conducted with stakeholders, including medical students and faculty from a private osteopathic school of medicine. Transcripts of these discussions were analyzed using Colaizzi’s method for qualitative analysis.

**Results:**

Five key themes emerged: (1) curricular gaps and structural barriers to comprehensive weight bias and obesity education, (2) impact of clinical exposure on reinforcing or challenging bias, (3) need for targeted communication and clinical skills training, (4) value of experiential and interactive learning modalities, and (5) empathy, bias awareness, and holistic understanding of obesity.

**Discussion:**

These findings highlight the need and desire for structured education on weight bias during medical school. Results also identified preferred educational features within an e-learning context that can deliver impactful and engaging education. Integrating targeted e-learning and experiential strategies into the medical school curriculum is a promising educational modality to improve core knowledge, build empathy, and improve communication techniques, promoting an inclusive, patient-centered focus among future physicians when treating patients with obesity.

## Introduction

Obesity is currently one of the most prevalent public health challenges in the United States, with over 40% of adults affected [1]. While there is a focus on expanding treatment options, rising obesity rates are accompanied by increased weight bias, defined as having negative attitudes toward and beliefs about others because of their weight [2]. Weight bias is prevalent in healthcare settings, where providers, including physicians, perpetuate weight bias [3, 4]. Patients who experience weight bias are more likely to avoid future care, report greater mistrust of physicians, and receive lower-quality care compared to patients with a lower body mass index [5–8].

Healthcare-related weight bias is not limited to practicing physicians; it is also common among medical students, driven by a variety of factors [9]. Despite the increased awareness of weight bias, obesity education in medical school is limited, with many schools reporting obesity education as a low priority [10]. Furthermore, medical students often report feeling uncomfortable having weight or obesity management-related discussions with patients [11]. Efforts to provide obesity education in medical school are often inadequate and lack a clearly defined approach to reducing weight bias among medical students [12, 13]. Additionally, it is unknown whether these efforts translate into clinical behavioral improvements. Advocates and researchers in the field believe that more rigorous and multi-modal approaches to obesity and weight bias education are needed and should be prioritized to both minimize adverse outcomes for patients and promote equitable healthcare practices [14, 15].

One possible approach to addressing the weight bias education gap is to integrate e-learning modules into medical school curricula. While e-learning is a well-documented component of medical education delivery, limited research exists on its use for weight bias instruction among medical school students [16, 17]. Thus, there is an opportunity to develop, disseminate, and evaluate weight bias education via e-module delivery. This study utilized focus groups of targeted stakeholders - medical students and medical school faculty - to help inform content for e-learning module development. This paper reports on the focus group findings, which served as scaffolding to develop weight bias e-learning modules for medical students as part of a larger project.

## Theoretical Framework

Implicit Bias Theory offers a valuable lens for understanding how weight bias emerges and operates within medical education and clinical practice. Implicit bias refers to the automatic, unconscious attitudes and stereotypes individuals hold toward others that shape perceptions, judgments, and behaviors, even when these attitudes conflict with consciously endorsed beliefs [18]. These associations are shaped by lived experiences, cultural messages, and broader societal norms, and they often influence interactions in subtle but meaningful ways.

Within medical settings, implicit weight bias can affect communication, clinical decision-making, and the overall quality of patient care [5–8]. Because these biases frequently operate outside conscious awareness, they may manifest through tone, body language, assumptions about patient behavior, or the framing of weight-related discussions. For medical students and faculty, recognizing how implicit processes may shape clinical encounters is a critical step toward fostering equitable, patient-centered care.

Using Implicit Bias Theory as a guiding framework helps contextualize the perspectives shared in our focus groups. Participants described how weight-related assumptions may influence patient interactions, how educational or clinical experiences shape their awareness of bias, and where gaps remain in current training. This theoretical grounding supports the need for intentional, structured weight-bias education within the medical school curriculum—education that addresses explicit attitudes and equips learners to identify and mitigate the subtle processes that influence clinical behavior. By situating the focus group findings within Implicit Bias Theory, this work highlights the importance of integrating bias-mitigation strategies into medical training and underscores the potential for such efforts to improve communication, enhance empathy, and ultimately strengthen patient care.

## Methods

### Setting

The research was conducted through a collaborative partnership between two institutions in southeastern Pennsylvania. Villanova University’s M. Louise Fitzpatrick College of Nursing, a private institution dedicated to advancing nursing education, research, and advocacy, served as the primary institution, research lead, and grant recipient. The Philadelphia College of Osteopathic Medicine (PCOM), which specializes in training osteopathic physicians, served as the study site. This partnership has been ongoing for several years, providing faculty and students with opportunities for interdisciplinary collaboration and education across health disciplines.

### Participants and Recruitment

Two stakeholder groups from the medical institution - students and faculty - were recruited as study participants. Second- and fourth-year medical students were invited via email, specifically because they would not be eligible for the e-learning educational series in future semesters. Faculty members teaching within the institution’s Interprofessional Education (IPE) curriculum were also recruited. Recruitment strategies for both groups included email communication, snowball sampling, and colleague referrals. Recruitment emails were sent by PCOM’s IPE Director, who oversees the curriculum where the e-learning modules would eventually be implemented. Over 200 medical students received recruitment information; although up to 10 students were sought, eight participated in the focus group sessions. Faculty recruitment aimed for 10 participants, with five ultimately agreeing to participate. The study was approved by Villanova University’s Institutional Review Board (IRB-FY2025-115). All participants provided informed consent, and medical students received monetary compensation via an electronic gift card at the conclusion of the focus groups.

### Design

A semi-structured focus group methodology was selected as the primary research approach, as it is commonly used in social science research and well-suited for small group settings. This qualitative method allowed participants to share their perspectives on obesity and weight bias content within the current medical school curriculum. Student focus group questions aimed to assess baseline knowledge of weight bias, identify gaps in the curriculum, explore differences between pre-clinical and clinical education, and understand how students learn most effectively from e-learning modules. Faculty, serving as content experts, provided insights on how obesity and weight bias are currently taught, as well as the barriers and opportunities for integrating these topics. Faculty focus group questions concentrated on identifying curriculum areas addressing weight bias and on features of e-learning modules that best support student learning and align with the existing curriculum. A discussion guide structured each focus group session; sample questions for both students and faculty are provided in Tables 1 and 2.

**Table 1:** Sample Semi-Structured Focus Group Questions for Students

Section 1: Experiences with Weight Bias and Obesity
Are you familiar with the term weight bias? How would you describe it? Have you learned about weight bias before in your medical school courses? Based on your experiences and what you know about weight bias, do you think this is an issue in healthcare?
Section 2: Medical School Education on Weight Bias and Gaps
Now that you are close to the end of your medical school education, what gaps if any do you see in the areas of weight bias or obesity education, including practical skills such as how to conduct sensitive weight-related conversations with patients? Is there any other training or education in these areas that you think would be helpful?
Section 3: Weight Bias IPE Program Planning/Content and Format Recommendations
[PCOM] has a very robust IPE program and a new 4-module self-paced pre-work series on weight bias is being developed as another offering within the program. What do you think are the most important topics you would want to see included in such a program? What professional or group of professionals would you think are most suited to deliver this training to medical students?

**Table 2:** Sample Semi-Structured Focus Group Questions for Faculty

Section 1: Medical School Education on Weight Bias and Gaps
1. Do you know of any areas in the curriculum where obesity education, and specifically weight bias, is addressed? If so, can you tell us how it was incorporated into the curriculum, delivered as a standalone offering, or as provided as a program outside of the curriculum? 2. Do you think the way obesity as a disease and weight bias is addressed in medical school prepares students well for conducting sensitive weight-related conversations in clinical practice? 3. From your perspective, what gaps, if any, in the curriculum do you see ​regarding​​ ​ obesity, weight-related health issues, and/or weight bias?
Section 2: Weight Bias IPE Program Planning/Content and Format Recommendations
[PCOM] has a very robust IPE program and a new 4-module virtual self-paced pre-work series on weight bias is being developed as another offering within the program. The series will focus on obesity education, including improving awareness and knowledge of weight bias in health care. 1. What skills do you believe are essential for addressing weight bias/obesity education in this module series? 2. What teaching methods do you think would be most effective in delivering content on weight bias​?​ 3. How can we best ensure that students are not only learning about weight bias but are also applying that knowledge in practice?

### Procedure

All research team members had experience in qualitative research and conducting focus groups. Each focus group was facilitated by two researchers: one serving as the primary interviewer and the other as the scribe, responsible for notetaking and managing the recording. Two focus groups were conducted with medical students in February 2025 (Second-year students, *n* = 4; Fourth-year students, *n* = 4), and two focus groups were conducted with faculty in March 2025 (Focus group 1, *n* = 3; Focus group 2, *n* = 2). Two faculty focus groups were held to accommodate scheduling constraints and ensure optimal participation. Each session lasted approximately 60 min and was conducted via Zoom, with audio recordings captured through the platform. To maintain confidentiality, participants were identified by numbers (e.g., ‘Student 1,’ ‘Faculty 1’) rather than by name. Following each session, research team members reviewed the transcripts for accuracy prior to beginning the formal thematic analysis.

### Data Analysis

Qualitative data were analyzed using Colaizzi’s phenomenological method to identify emerging patterns [19]. Student and faculty responses were coded and analyzed separately. Two research team members independently reviewed the data and identified initial themes for each guiding question. Recurring ideas were compared to ensure that each category accurately reflected participants’ perspectives. This iterative process continued until all relevant data were categorized and significant statements extracted. The research team then reviewed the summarized data, finalized themes for each group, and ensured clarity and accuracy in the narrative descriptions. Primary themes were reported as aggregate findings for each participant group and compared across groups to identify similarities and differences. These findings informed the development and implementation of four e-learning modules within the medical school curriculum, providing critical insights into students’ educational needs and faculty perspectives.

## Results

The thematic analysis of the focus group transcripts yielded 58 significant statements from the student focus groups and 51 from the faculty focus groups. From this analysis, five primary themes emerged that highlighted the critical gaps, barriers, and opportunities in the current curriculum and future educational endeavors: (1) curricular gaps and structural barriers to comprehensive weight bias and obesity education, (2) impact of clinical exposure on reinforcing or challenging bias, (3) need for targeted communication and clinical skills training, (4) value of experiential and interactive learning modalities, and (5) empathy, bias awareness, and holistic understanding of obesity.

### Theme 1: Curricular Gaps and Structural Barriers to Comprehensive Weight Bias and Obesity Education

#### Shared Insight

Students and faculty acknowledge a lack of consistent, integrated, and adequately resourced education on obesity and weight bias across the curriculum. This gap limits learners’ opportunities to develop the skills and confidence needed to address weight-related concerns in clinical practice.

#### Student Perspective

Students noted that weight bias education is often incidental rather than embedded in formal coursework, leaving them unprepared to initiate and guide sensitive conversations about weight with patients. Several participants emphasized that medical education may inadvertently introduce, normalize, or even reinforce weight bias through diagnostic framing, for example, presenting obesity primarily as a personal failure or lifestyle choice rather than a complex, multifactorial chronic disease. Additionally, case studies and clinical scenarios often lack representation of diverse body sizes, reinforcing narrow assumptions about health and appearance. Students expressed that direct interaction with patients of varied body sizes, combined with structured training, could help them develop more inclusive attitudes and communication strategies to approach weight-related topics with sensitivity and respect.

Student 6: *“I don’t know if you all heard of this mnemonic for cholecystitis*,* is ‘female fat 40.’ So….when we see that gender*,* that age group*,* and*,* hinting at a high BMI*,* the question stems automatically. So reflecting back*,* I feel like that’s how we’re being trained to have sort of biases*,* and to think a different way*,* whenever we see an overweight patient or obese patient.”*

#### Faculty Perspective

Faculty highlighted logistical, financial, and structural barriers to integrating weight bias education into the medical curriculum, noting these challenges often lead to inconsistent implementation. They emphasized the need for a more standardized, well-supported approach to ensure students receive comprehensive training in addressing weight bias and obesity care in general. Faculty also recognized that medical students would benefit from exposure to diverse professional pathways and specialties involved in weight management to broaden their understanding of what it means to be an obesity or weight management specialist.

Faculty 1: *“There are definitely gaps in the curriculum when it comes to obesity. It is generally referred to in the context of BMI as a risk factor for other conditions like cardiovascular disease*,* diabetes*,* cancer*,* or liver disease*,* but it is not addressed as a specific entity. To my knowledge*,* weight bias is not addressed in the curriculum at all.”*

### Theme 2: Impact of Clinical Exposure on Reinforcing or Challenging Bias

#### Shared Insight

Clinical environments play a critical role in shaping attitudes toward weight, as they serve as influential settings where students and clinicians observe, internalize, and model professional behaviors toward patients with diverse body sizes. These clinical environments often reinforce implicit and explicit norms - through provider communication, institutional practices, and the prioritization of specific health indicators - that can either perpetuate or challenge weight-biased beliefs.

#### Student Perspective

Students recognized that biased attitudes and behaviors are often learned and modeled after others in supervisory positions and in team dynamics. In clinical environments, students witnessed weight bias being expressed both overtly and subtly, and exposure to poor role models contributes to internalization of biased attitudes and behaviors as acceptable or normative.

Student 2: “*I’m gonna take the not-so-nice approach to this*,* what I’ve seen on the floor and in the ER. It’s a very negative perception on [obesity] being a hassle on the system for one reason or another. Either it takes more time*,* or manpower*,* more finances to work with a patient who may have a higher body habitus. It creates this almost negative stigma*,* [and] negative atmosphere all across the healthcare team*,* especially if the person that starts to embed that attitude is one of the higher ups like the doctor. And then trickles down*,* and that’s where I saw it the most*,* especially in the ER*,* as soon as the attending said something*,* everybody else kind of jumped on board with that mindset.”*

#### Faculty Perspective

Faculty acknowledged that clinical training must include intentional strategies to counteract weight bias and promote equitable, patient-centered care. From their perspective, students often enter clinical settings without the skills to recognize or challenge biased remarks or practices modeled by staff. Faculty emphasized the importance of integrating structured learning and reflection into clinical rotations so students can critically assess what they observe and develop professional behaviors that support respectful, inclusive care for patients of all body sizes.

Faculty 4: *“I think about our clinic- and some of the most awkward and challenging times happen before we even start the conversation. Let’s say someone needs an oversized wheelchair*,* and we can’t get them in comfortably*,* and it’s already awkward for them. We haven’t done anything yet*,* but it’s already stressful*,* and I feel stressed*,* they feel stressed.”*

### Theme 3: Need for Targeted Communication and Clinical Skills Training

#### Shared Insight

Effective communication about weight requires guided, practical training that builds students’ skills and confidence. Without directed instruction, students may unknowingly default to stigmatizing language or avoid the topic altogether. Integrating this type of education into coursework and clinical experiences supports respectful, patient-centered conversations about weight.

#### Student Perspective

Students discussed feelings of discomfort and uncertainty when discussing weight with patients, especially when they lack adequate training and patients do not perceive weight as a concern. Discomfort or fear of offending patients also prevents students from initiating weight-related conversations. A lack of consistent communication and role modeling by providers in clinical settings made it difficult for medical students to acquire-and practice-sensitive weight-related communication skills.

Student 7: *“I feel like weight was more of a topic that it depended on the provider in terms of how they approached it. Some of them would never bring up weight or weight loss or strategies because they felt like it was too sensitive of a topic for patients that were overweight.”*

#### Faculty Perspective

Faculty voiced support of active learning methods (e.g., simulation, role play, motivational interviewing practice sessions) to build competency in sensitive weight-related conversations. They advocated for practiced training with specific guidance (tool/script) on how to discuss weight sensitively and effectively best to improve student confidence and consistency in patient interactions.

Faculty 1: “*I wonder if there’s a script [for] taking a weight or obesity type history. We can teach a student how to ask about smoking*,* and how many years they smoked*,* and when they gave it up? But I wonder if there is a template that we can teach the students*,* especially early on in their 1st or 2nd years. [We] have them ask about a sexual history*,* but I don’t know if we know how to ask them about their diet or their weight. That kind of framework*,* and medical students*,* in my opinion*,* are very good. If you tell them this is what you have to do*,* and if you can find an acronym for it*,* you’ll even be better.”*

### Theme 4: Value of Experiential and Interactive Learning Modalities

#### Shared Insight

Both groups favored interactive, engaging educational strategies over passive instruction. This theme highlighted that moving beyond traditional textbook learning through engagement and interaction can improve and enhance the learning experience.

#### Student Perspective

Students valued practice opportunities to apply concepts in real or simulated settings, opportunities for self-reflection to recognize personal biases, and short, engaging modules with videos and interactive questions. Also, knowing that content will be discussed or evaluated later increases its perceived value.

Student 5: *“I think*,* having a definition of weight-based bias is really important. And having examples of what weight bias is*,* and also*,* how can it affect patients*,* [and] what are some of the patient experiences*,* I think*,* even more impactful*,* and then having tools that we can actually use in clinical practice as to how can we avoid weight bias.”*

#### Faculty Perspective

Faculty believed that teaching modalities such as simulation and role-play are impactful methods for teaching empathy and bias awareness. In addition, any learning style that involves peer observation and/or feedback enhances skill development and reflective practice. Similarly to students, faculty agreed that students are more likely to engage with content that is assessed or relevant to the medical boards.

Faculty 3: *“I do feel ….actual interaction with patients tends to be something that students enjoy most*,* because*,* like*,* eventually*,* that is their goal.”*

### Theme 5: Empathy, Bias Awareness, and Holistic Understanding of Obesity

#### Faculty Perspective Only

Unlike other themes that were shared by both faculty and students, this theme emerged exclusively from faculty discussions. Faculty expressed concern that medical students often demonstrate a lack of empathy toward patients with obesity and tend to hold overly simplistic or biased views of obesity as a disease. This observation was not mirrored by students, who did not self-identify as lacking empathy; rather, students more commonly described witnessing weight bias or limited empathy among supervisors or within clinical environments. Faculty emphasized the importance of addressing unconscious bias through self-reflection, understanding social determinants of health, challenging myths and misconceptions, and fostering a more compassionate, nuanced approach to care. For example, using patient narratives and stories from providers and/or patients with lived experience of overweight and obesity to humanize the issue, move beyond judgment, and illustrate the real-world impact of weight bias.

Faculty 4: “*I think*,* in general*,* we could do better. One thing I’ve noticed in some medical students is that they don’t have enough empathy for the people in front of them*,* including*,* and especially*,* individuals with obesity. And I think there could be initiatives done in different ways that might be able to kind of pull that empathy out. One is that I think there’s not enough connection between their understanding of their own beliefs and attitudes*,* and even behavior*,* and how it actually relates to outcomes in individuals with obesity. I don’t think they realize what impact they could have.”*

## Discussion

This study examined the perspectives of medical students and faculty on weight bias in the broader context of obesity education in the medical school curriculum through semi-structured focus group interviews. Thematic analysis revealed five key themes that highlighted significant gaps and opportunities to enhance current educational practices. These themes inform the following suggestions for building effective and sustainable educational opportunities for students.

### Curricular Gaps and Structural Barriers to Comprehensive Weight Bias and Obesity Education

Student and faculty participants consistently identified a lack of structured, longitudinal integration of obesity and weight bias content across the curriculum. Instruction on these topics was often limited to electives or isolated lectures, with little consistency across the preclinical and clinical years. These findings align with prior research indicating a striking paucity of obesity and weight bias related content in undergraduate medical education, both in terms of content and clinical exposure [7, 10, 12, 20]. Moreover, logistical and financial barriers, such as limited simulation lab access and a lack of student and faculty time, were cited as significant obstacles to implementing more robust educational interventions. These concerns mirror the frequently mentioned barriers to adequate time allocation for obesity and weight bias education in medical training [10, 12, 20, 21]. However, evidence supports early and consistent integration of education throughout medical training. The medical training years are frequently viewed as a ripe time to mold students’ beliefs and attitude formation, to encourage an openness to transformative insights, and promote an increased awareness and foundational knowledge around obesity and biases [21–23]. Obesity, and specifically weight bias education, continue to be marginalized in medical training. Moving towards a comprehensive curriculum requires time, institutional commitment, faculty expertise, and even national policy alignment to ensure equity and sustainability in teaching providers to care for patients with obesity effectively. However, the potential benefits in improved patient health outcomes and quality of provider-patient communication regarding weight are notable.

### Impact of Clinical Exposure on Reinforcing or Challenging Bias

Both students and faculty recognized the influential role of clinical environments in shaping attitudes toward weight. Students reported observing both overt and subtle expressions of weight bias during clinical training, potentially internalizing these attitudes through exposure to poor role models and team dynamics. This underscores the importance of addressing bias not only in formal education but also within the hidden curriculum of clinical practice. The hidden curriculum refers to the idea of implicit lessons, values, and norms that medical students absorb through observation, institutional culture, and informal interactions, which can significantly shape attitudes toward patients with obesity [24–27]. These unspoken influences often contradict formal teachings that emphasize empathy, patient-centered care, and evidence-based practice. Within clinical environments, students may witness physicians and other healthcare professionals dismissing patient concerns, attributing symptoms solely to weight without adequate investigation, using stigmatizing language, or observe patients with obesity becoming the subject of derogatory humor [28]. Although these behaviors are not part of the formal curriculum, they are often internalized by students as acceptable or routine, making it less likely that they will challenge such biases [27]. This normalization of weight bias within the clinical setting can negatively affect how future physicians engage with patients with obesity. It may diminish the quality of patient-centered behaviors such as friendliness, attentiveness, respectfulness, and meaningful interaction [27]. Faculty also acknowledged this challenge and emphasized the need for intentional strategies within clinical training to counteract bias and foster empathy. These insights underscore the need for students and faculty alike to recognize factors that may perpetuate bias and stigma in the classroom and clinical environment and focus on practices and education that exemplify inclusive, patient-centered care.

### Need for Targeted Communication and Clinical Skills Training

A clear need for targeted communication and clinical skills training emerged as a central theme. Participants emphasized the importance of explicit instruction on how to engage in sensitive conversations about weight and obesity. Medical students frequently reported discomfort and uncertainty when initiating these discussions, often citing a lack of structured tools, language guidance, or practice opportunities, which parallels the literature on this topic [1, 10, 11, 29]. Faculty also advocated for the development and integration of active learning strategies like simulation-based activities, role-playing, and motivational interviewing practice to help students build confidence and competence in these interactions. Incorporating proficiency-based elements, such as simulated patient encounters and communication skill assessments, allows students to demonstrate mastery through practical application. These experiential approaches reinforce technical communication skills, promote empathy, and reduce bias. Evidence supports the effectiveness of such methods in improving provider-patient interactions and fostering more respectful, inclusive care for patients with obesity [30, 31]. By embedding these strategies into the curriculum, medical education can better prepare students to navigate complex conversations with sensitivity, professionalism, and confidence.

### Value of Experiential and Interactive Learning Modalities

Both students and faculty valued moving beyond passive, lecture-based instruction toward more interactive and experiential learning approaches. Participants consistently expressed that traditional textbook-driven methods often fail to engage learners or foster meaningful understanding, particularly when addressing complex and sensitive topics like weight bias and obesity care. Instead, they advocated for educational strategies that actively involve learners through dynamic modalities such as videos, interactive case studies, reflective exercises, and real or simulated clinical practice. Students voiced a strong preference for short, focused modules that incorporate opportunities for self-reflection, allowing them to identify and confront personal biases. They also valued clear connections between learning activities and future assessments or clinical applications, which helped reinforce the relevance of the content, as previously discussed. Faculty noted that techniques like simulation-based activities, role-playing, and peer feedback are particularly effective in cultivating empathy and bias awareness. These methods enhance engagement and create safe spaces for learners to practice and refine communication and clinical skills. Faculty highlighted that students are more likely to engage with content that is either assessed or aligned with licensure exam preparation. This insight underscores the need to integrate experiential components into the formal curriculum in ways that are pedagogically sound and strategically positioned within existing assessment frameworks. Together, these perspectives support the development of educational modules that are interactive, reflective, and assessment-driven to improve learning outcomes and promote bias reduction [32, 33].

### Empathy, Bias Awareness, and Holistic Understanding of Obesity

While skill-building in communication is essential, faculty emphasized that technical proficiency alone is insufficient to address the deeper attitudinal challenges surrounding obesity care. Beyond structured training, there is a pressing need to cultivate empathy, raise awareness of implicit bias, and promote a more holistic understanding of obesity among medical students. This theme, drawn primarily from faculty perspectives, stresses the importance of shaping not just what students do, but how they think and feel about patients with obesity.

Faculty consistently expressed concern over the limited empathy and binary thinking students often exhibit when discussing patients with obesity. These attitudes may be shaped by the hidden curriculum or by societal norms, and reinforced within clinical environments, and can lead to oversimplified views of health and body size, where obesity is seen solely as a personal failure or a risk factor to be corrected. Such perspectives overlook the complex interplay of biological, psychological, social, and environmental factors that contribute to obesity. To counter these reductive views, faculty advocated educational strategies that have been discussed throughout this paper, such as self-reflection and exercises that challenge assumptions. Furthermore, incorporating patient narratives, reflective writing, and facilitated discussions with providers and patients living with obesity were seen as tools for advancing empathy and bias awareness. Key features of impactful weight bias education, such as reflective journaling, have been shown to increase bias awareness in students [34, 35]. Moreover, faculty emphasized the importance of teaching students to view obesity holistically, recognizing it as a chronic, multifactorial condition rather than a simple outcome of lifestyle choices. This shift in perspective is critical for developing compassionate, evidence-informed practices that respect patient autonomy and dignity.

### Limitations

This study has several limitations for consideration. First, using a convenience sample from a single osteopathic medical school limits the generalizability of the findings to other medical schools, particularly those with different curricular structures or cultural contexts. The small sample sizes may not fully capture the diversity of perspectives within medical education. it is possible that the focus group design may naturally introduce social desirability bias, as discussions centered on sensitive topics such as bias and empathy. Lastly, some focus group questions may not have been as open-ended as intended, which could have influenced the nature of participants’ responses.

### Future Directions

Building on these findings, future research should evaluate the impact of structured weight bias education on learner outcomes and observed patient care. Longitudinal studies are needed to assess whether interventions such as e-learning modules lead to sustained improvements in attitudes, communication skills, and clinical behaviors. E-learning offers flexibility, scalability, and the ability to incorporate interactive elements such as videos, quizzes, and reflective exercises that promote deeper engagement. These modules can serve as a consistent and accessible tool for reinforcing bias awareness and clinical competencies across diverse learning environments. Additionally, expanding this work to include interprofessional education and diverse healthcare settings would provide a more comprehensive understanding of how to reduce weight bias across the continuum of care. From a practice standpoint, medical schools should consider embedding weight bias education into competency-based assessments and licensure examination-relevant content to ensure engagement and accountability. Ultimately, aligning educational strategies with institutional priorities and national standards will be critical for promoting equitable, patient-centered care for individuals living with obesity.

## Conclusion

This study revealed critical insights into how weight bias is perpetuated and can be addressed within medical education. Through focus group discussions with medical students and faculty, several interconnected themes emerged: structural and curricular gaps in obesity education, the influence of clinical exposure on bias formation, the need for targeted communication and clinical skills training, the value of experiential learning, and the importance of empathy building, bias awareness, and a holistic understanding of obesity. The findings provide a strong foundation for the development of relevant educational content to address weight bias in medical training. The identification of curricular gaps and emphasis on communication and clinical skills training suggest valuable components such as case-based scenarios, simulation-based activities, and patient-centered interviewing techniques. These elements can help students practice sensitive conversations and build confidence in patient interactions. Additionally, the theme of empathy and holistic understanding calls for the inclusion of patient narratives, guided reflection exercises, and content that explores the complex, multifactorial nature of obesity beyond simplistic or stigmatizing frames. Such educational content has the potential to mitigate the impact of the hidden curriculum, promote inclusive and respectful care, and prepare future physicians to engage with patients with obesity in a manner that is clinically effective and ethically grounded. Addressing weight bias through thoughtful, evidence-informed education is a critical step toward advancing equity and compassion in healthcare.
